# A Case Report of Perceptual Disturbances with Incidental Calcifications in the Cerebellum

**DOI:** 10.1155/2021/2680674

**Published:** 2021-09-29

**Authors:** Sarah Manzoor, Lillian Sangha, Pavandeep Badh, Heela Azizi, Ayodeji Jolayemi

**Affiliations:** ^1^Department of Psychiatry, Interfaith Medical Center, American University of Antigua College of Medicine, Brooklyn, New York, USA; ^2^Department of Psychiatry, Interfaith Medical Center, Medical University of the Americas, Brooklyn, New York, USA; ^3^Department of Psychiatry, Interfaith Medical Center, Brooklyn, New York, USA

## Abstract

**Background:**

The cerebellum has extensive connections with motor and nonmotor areas of the nervous system. These nonmotor areas include the cognitive, affective, and perceptual areas of the central nervous system. Extensive literature has emerged cognitive documents and mood disorders in patients with cerebellar dysfunction. Perceptual disturbances consistent with cerebellar connections with perceptual areas have not been as widely documented. *Case Presentation*. We present the case of a 58-year-old female presenting with new onset isolated auditory hallucinations and incidental findings of cerebellar calcifications.

**Conclusion:**

In light of this case, we discuss an expanding body of evidence that suggests the likely role of the cerebellum in perceptual functioning.

## 1. Introduction

The cerebellum comprises about 10% of the mass of the brain but contains approximately 50% of all neurons in the brain [1]. The cerebellum is divided into different regions: vestibulocerebellum, spinocerebellum, and cerebrocerebellum. The vestibulocerebellum plays a role in gross and fine movement. The spinocerebellum affects accurately coordinated movements interfering with movement regulation. The cerebrocerebellum plays a role in planning movements and sensory input, affecting reaction time [1]. The cerebellum plays a key role in sensory-motor pathways; however, increasing evidence of its role in cognitive functions has emerged. Recent imaging from functional MRI studies has mapped cognitive functions to a lateral cerebellar distribution. Specifically, the neocerebellum and ventral lateral aspects of the dentate nuclei are responsible for the cognitive functions of the cerebellum. In addition, language-related functions have been mapped to the right posterior cerebellar hemisphere in individuals who are left cerebral hemisphere language dominant. Visuospatial function lateralizes to the left posterior hemisphere. Executive functions are notably bilateral while affective functions are primarily midline in the limbic cerebellum [2].

Neuroimaging studies showed that the lateral hemispheres of the posterior cerebellum (lobules VI-IX) and flocculonodular lobe (lobule X) were associated with cognitive function while the anterior lobe (lobules I-V) was primarily responsible for motor function [2].

Consistent with functional MRI findings of these nonmotor functions is clinical evidence revealing how cerebellar pathology is associated with cognitive and psychiatric illnesses such as “dysmetria of thought,” bipolar disorder, PTSD, attention deficit, autism spectrum disorders, and schizophrenia [2].

For over three decades, clinical, experimental, and neuroimaging studies have shown strong evidence for the involvement of the cerebellum in diverse areas of cognition such as memory, language, and emotion. Several studies demonstrate that the cerebellum affects perception [3]. Anatomical and electrophysiological studies that have involved monkeys and cats indicate the existence of cerebellar connections between visual- and auditory-related cortices. Furthermore, case reports on humans have elicited that both focal and diffuse lesions of the cerebellum lead to an array of sensory dysfunctions such as time and motion perception or recognition of perceptual sequences [3]. Research involving healthy controls as well as patients with cerebellar disorders has indicated that several neurological conditions in which perceptual disturbances occur, including autism and schizophrenia, are associated with cerebellar pathology [3]. This supports the concept that cerebellum is an essential neural circuit involved in sensorimotor, autonomic, and cognitive function.

Researchers have proposed various hypotheses linking cerebellar function to perception. One main hypothesis is that the cerebellum is responsible for information processing by making predictions in the form of an “internal model” of sensory events. Another explanation is that the cerebellum facilitates perception by monitoring and coordinating the acquisition of sensory information [3]. Furthermore, it has been hypothesized that the cerebellum functions as an internal timing device for both motor and perceptual processes. This includes different regions of the cerebellum providing separate timing computations for different tasks. At the moment, there is no clear support for any of the mentioned theories, but they all individually contribute to the present understanding of the function of the cerebellum [3].

## 2. Case Presentation

We present the case of a 58-year-old Guyanese-Indian female who presented to the psychiatric emergency room after she called 911 regarding harassment from “an agency” that threatened her and her mother in their apartment. The patient stated that she called the police because the voices were emotionally distressing to her mother. In 2014, the patient transitioned from being a certified nurse assistant to a home health aide for her mother, which was a significant stressor. In 2019, she lost her insurance and was subsequently terminated from her job as a home health aide, but still continued to care for and live with her mother without pay. This event was another stressor that may have contributed to the escalation of the voices. The voices first began in 2014 and progressively increased in frequency.

The patient reported hearing approximately 5 different voices, male and female. She stated that they were subtle and had learned to ignore them. She denied that the voices were familiar and stated that the voices criticized and belittled her. The patient also believed that technology played a major role in the “agency's” ability to communicate with her. She believed there was a chip/camera in the house that was watching and communicating with her. The patient reported that whenever she opened the refrigerator, turned on the faucet, or turned on the air conditioner, the recordings were amplified. Most of the patient's auditory hallucinations occurred in her apartment, but other instances occurred on a bus, while she was undergoing an X-ray, and at multiple subway stations. The patient filed 10 police reports complaining of harassment. The patient recorded the voices she heard on her phone; however, she refused to share these recordings with the psychiatry team as she believed the situation would be better handled by law enforcement.

The patient had no prior history of any medical condition, diagnosed mental illness, abuse, head trauma, or prior history of admission to a psychiatric unit. She was not taking any medications. Her basic metabolic panel, coagulation profile, and urine toxicology were all within normal limits. She scored 30/30 on the Mini Mental Status Examination.

**Figure 1:**
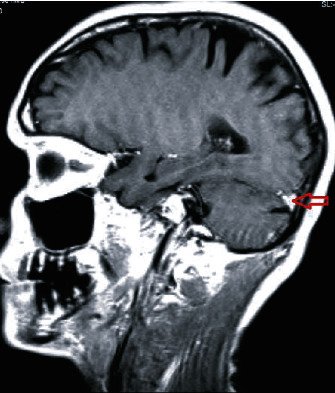
Patient MRI in sagittal view. Demonstrates calcifications in the supratentorial cerebellum.

**Figure 2:**
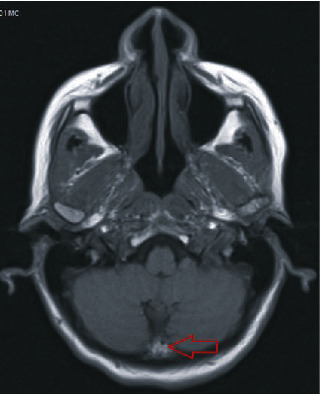
Patient MRI in axial view. Demonstrates calcifications in the supratentorial cerebellum.

The MRI of the head revealed an 8 × 6 mm extra-axial calcified lesion at the insertion of the left tentorium cerebelli in the left parietal calvarium, which suggests a calcified meningioma (Figures 1 and 2). The MRI indicated calcifications in the supratentorial cerebellum.

The psychiatry team explained to the patient that the voices she heard could be auditory hallucinations due to stress and that medication may help eliminate the voices. She denied medication, had no insight, and believed that the voices were real because of her experiences outside of the apartment and because her mother heard them as well. Since both the patient and mother claim to hear the audio recordings, it is a shared delusion or “folie à deux.” Family members reported to the psychiatry team that despite her auditory hallucinations, she was functional and her current state had been her baseline since 2014. The patient denied all medications and follow-up appointments with outpatient psychiatrists. We discussed various treatment modalities with the patient, including the risks and benefits associated with each. This included pharmacological treatment, cognitive behavioral therapy (CBT) with reality testing, and coping with perceptual disturbances. The patient opted for CBT and showed considerable improvement in coping with auditory hallucinations.

## 3. Discussion

The patient discussed in this case presented with auditory hallucinations that have been active for the past five years. These hallucinations have been progressively increasing in frequency, duration, and volume, and as a result, have caused distress. Schizophrenia was ruled out in this patient because she did not meet the diagnostic criteria beyond the presence of auditory hallucinations. In addition, there were no illicit substances, metabolic derangement, or other organic causes. A brain image with an 8 × 6 mm extra-axial calcified lesion at the left tentorium cerebelli, in the left parietal calvarium, which is suggestive of a calcified meningioma was the only positive finding. The absence of any significant medical or psychiatric history suggests that the calcifications found on neuroimaging studies may play a role in the development of this patient's auditory hallucinations. The incidental finding of seemingly isolated cerebellar pathology with the emergence of auditory hallucinations is consistent with the emerging literature on the perceptual role of the cerebellum.

Multiple studies have been done, highlighting this contribution within the auditory circuit, specifically within the cortico-ponto-cerebellar pathway [4]. A meta-analysis performed by Petacchi et al. showed that in response to auditory stimuli, the temporal auditory areas of the cerebral cortex displayed the most activation [5]. Certain cerebellar areas, however, particularly the left lateral crus I area of the left hemisphere, showed to have the next highest activation [5].

In addition, several studies have demonstrated that the cerebellum plays a role in the sensory processing of stimuli, specifically in regulating the input and evaluation of various sensory data [1]. The medial geniculate body of the thalamus relays information from the deep nuclei of the cerebellum to the secondary auditory cortex for sound identification [4]. This displays cerebellum's role in higher cortical function and supports that any disruption of auditory processing, including calcifications, has the potential to alter incoming sensory information. The Purkinje cells and deep nuclei of the cerebellum function to regulate incoming information. When this process is disrupted or altered in any way, the cerebellum may mistake an internal stimulus from the auditory cortex for an external signal, and as a result, the outgoing signal from the cerebellum to the cortex may be altered, potentially leading to an auditory hallucination [4]. Repetitive erroneous stimuli from the cerebellum to the cortex can cause hallucinations [4].

Several other case studies have also shown similar processes in which alterations to the cortico-ponto-cerebellar pathway can result in symptoms of psychosis, including auditory hallucinations [5–7]. Further research is strongly encouraged to fully understand the pathogenesis of cerebellar calcification-induced auditory hallucinations. A great understanding of the cerebellum will help to prevent pathological manifestations and potentiate treatment modalities.

Several case reports and case series in the past have linked cerebellar gross abnormalities to psychosis. A variety of pathologies have been implicated. A literature search in PubMed database revealed 21 studies associating Dandy Walker Complex involving the cerebellum with psychotic features [8, 9]. Associations between cerebellar stroke and psychosis have also been revealed in two recently published case reports [10, 11]. Cerebellar tumors [12, 13] and other cerebellar abnormalities [14, 15] have been similarly linked to psychosis. Among these studies, delusions were the most prevalent of the psychotic symptoms, followed by disorganized behavior, hallucinations, and disorganized thoughts, respectively.

An improvement in neuroimaging data, specifically with positron emission tomography (PET) and functional magnetic resonance imaging (fMRI), has indicated that the left Crus I area in the lateral cerebellum is consistently activated in brain imaging studies involving auditory tasks ranging from passive listening to pure tones or clicks [1]. Tasks involving temporal processing of auditory stimuli have shown that individuals with lateral cerebellar lesions could not accurately perceive the difference between “longer” and “shorter” acoustic tone bursts, and cerebellar involvement in this task was later confirmed in normal individuals in an fMRI study. Further analysis was done to determine if this activation was affected by attention, and it was ruled out that the cerebellar activation was primarily due to attentional demand [1]. In addition, several other studies have also shown the lateral cerebellum's involvement in speech stimuli on the basis of temporal dynamics.

## 4. Conclusions

There is significant evidence that the cerebellum contributes to auditory and visual sensory processes, but the exact functionality is not fully understood. Future studies are needed to identify functional imaging or PET scans of the cerebellar functioning in patients experiencing auditory hallucinations. It is important to understand how the cerebellum interacts with visual and auditory networks, especially regarding the nature (inhibitory or excitatory) and directionality (feedback or feedforward) of these connections. The pathology in the patient case demonstrates an association between cerebellar function and sensory perception which led to auditory hallucinations in our patient. There is increased evidence of mood and cognitive abnormalities in addition to the established motor function associated with the cerebellum. We are adding to the literature in perceptual abnormalities associated with the cerebellum.

The original understanding of the cerebellum as a fundamental motor functioning area of the brain has shifted with the increasing appreciation of its nonmotor function. Mood and cognitive cerebellar functions have been reported extensively, but little discussion about its perceptual function exists. Future studies are needed to explore the perceptual role of the cerebellum.

## Abbreviations

PTSD: Posttraumatic stress disorder
CBT: Cognitive behavioral therapy
PET: Positron emission tomography.

## Supplementary Materials

Supplementary Materials CARE checklist. This checklist is relevant to case reports and case series and is based on the CARE guidelines.
