# Supplementary material for: A Case Report of Perceptual Disturbances with Incidental Calcifications in the Cerebellum
Source: Case Rep Psychiatry. 2021 Sep 29;2021:2680674. doi: 10.1155/2021/2680674 (PMC8494594; doi:10.1155/2021/2680674)
Supplement: Supplementary Materials — CARE checklist. This checklist is relevant to case reports and case series and is based on the CARE guidelines. [file 2680674.f1.pdf]

CARE Checklist (2013) of information to include when writing a case report

CARE case report guidelines

(cc) BY-NC-ND

Topic	Item	Checklist item description	Reported on Page
Title	1	The words "case report" should be in the title along with the area of focus	Title: Page 1
Key Words	2	2 to 5 key words that identify areas covered in this case report.	Keywords: Page 1
Abstract	3a	Introduction—What is unique about this case? What does it add to the medical literature?	Abstract, Background, Page 2
	3b	The main symptoms of the patient and the important clinical findings	Abstract, Case Presentation, Page 2
	3c	The main diagnoses, therapeutics interventions, and outcomes	Case Presentation, Page 4 & 5
	3d	Conclusion—What are the main "take-away" lessons from this case?	Abstract, Conclusion, page 2
Introduction	4	One or two paragraphs summarizing why this case is unique with references	Introduction, page 3 & 4
Patient Information	5a	De-identified demographic information and other patient specific information	Case Presentation, page 4, paragraph 1
	5b	Main concerns and symptoms of the patient	Case Presentation, page 4
	5c	Medical, family, and psychosocial history including relevant genetic information (also see timeline).	Case Presentation, Page 4
	5d	Relevant past interventions and their outcomes	Case Presentation, Page 4, Paragraph 3
Clinical Findings	6	Describe the relevant physical examination (PE) and other significant clinical findings.	Case Presentation, Page 4 & 5, Paragraph 3 & 4
Timeline	7	Important information from the patient's history organized as a timeline	Case Presentation, Page 4& 5
Diagnostic Assessment	8a	Diagnostic methods (such as PE, laboratory testing, imaging, surveys).	Case Presentation, Page 5
	8b	Diagnostic challenges (such as access, financial, or cultural)	N/A
	8c	Diagnostic reasoning including other diagnoses considered	Discussion & Conclusion, Page 7
	8d	Prognostic characteristics (such as staging in oncology) where applicable	N/A
Therapeutic Intervention	9a	Types of intervention (such as pharmacologic, surgical, preventive, self-care)	Case Presentation, Page 5 & 6, paragraph 5
	9b	Administration of intervention (such as dosage, strength, duration)	N/A
	9c	Changes in intervention (with rationale)	N/A
Follow-up and Outcomes	10a	Clinician and patient-assessed outcomes (when appropriate)	Case Presentation, Page 5 & 6, paragraph 5
	10b	Important follow-up diagnostic and other test results	N/A
	10c	Intervention adherence and tolerability (How was this assessed?)	N/A
	10d	Adverse and unanticipated events	N/A
Discussion	11a	Discussion of the strengths and limitations in your approach to this case	N/A
	11b	Discussion of the relevant medical literature.	Discussion & Conclusion, Page 6 & 7
	11c	The rationale for conclusions (including assessment of possible causes)	Discussion, Paragraph 1, Page 6
	11d	The primary "take-away" lessons of this case report	Discussion & Conclusion, Page 7 & 8
Patient Perspective	12	When appropriate the patient should share their perspective on the treatments they received	N/A
Informed Consent	13	Did the patient give informed consent? Please provide if requested	Yes ☑ No ☐
